# Effect of inactivated COVID-19 vaccines on seizure frequency in patients with epilepsy: A multicenter, prospective study

**DOI:** 10.3389/fimmu.2022.984789

**Published:** 2022-12-07

**Authors:** Xiqin Fang, Shimin Hu, Tao Han, Tingting Yang, Junji Hu, Yucheng Song, Chunxiang Li, Aihua Ma, Yufeng Li, Qingxia Kong, Liou Tang, Wei Chen, Wenxiu Sun, Chunyan Fang, Yanping Sun, Juan Chen, Wenying Sun, Yibing Yan, Yuxing Gao, Jianhong Geng, Nan Li, Qiubo Li, Zhaolun Jiang, Shishen Lv, Wenke Li, Xiaoling Lang, Suli Wang, Yanxiu Chen, Baomin Li, Ling Li, Xinjie Liu, Yong Liu, Yan Zhan, Zaifen Gao, Lixin Qu, Qingxi Fu, Xuewu Liu

**Affiliations:** ^1^ Department of Neurology, Qilu Hospital, Shandong University, Jinan, China; ^2^ Institute of Epilepsy, Shandong University, Jinan, China; ^3^ Department of Neurology, Xuanwu Hospital, Capital Medical University, Beijing, China; ^4^ Beijing Key Laboratory of Neuromodulation, Beijing, China; ^5^ Institute of Sleep and Consciousness Disorders, Center of Epilepsy, Beijing institute for Brain Disorders, Capital Medical University, Beijing, China; ^6^ Department of Neurology, Shandong Provincial Hospital affiliated to Shandong First Medical University, Jinan, China; ^7^ Department of Neurology, Zibo Changguo Hospital, Zibo, China; ^8^ Department of Neurology, Jining City Dai Zhuang Hospital, Jining, China; ^9^ Department of Pediatrics, Yantai Yuhuangding Hospital, Yantai, China; ^10^ Department of Pediatrics, Shandong Provincial Hospital affiliated to Shandong First Medical University, Jinan, China; ^11^ Department of Pediatrics, Linyi People’s Hospital, Linyi, China; ^12^ Department of Neurology, Affiliated Hospital of Jining Medical, Jining, China; ^13^ Department of Neurology, Affiliated Hospital of Qingdao University, Qingdao, China; ^14^ Department of Neurosurgery, Liaocheng People’s Hospital, Liaocheng, China; ^15^ Department of Neurology, Zhucheng People’s Hospital, Zhucheng, China; ^16^ Department of Neurology, Heze Third People’s Hospital, Heze, China; ^17^ Department of Pediatrics, Liaocheng People’s Hospital, Liaocheng, China; ^18^ Department of Neurosurgery, The First Affiliated Hospital of Shandong First Medical University, Jinan, China; ^19^ Department of Neurology, Affiliated Hospital of Weifang Medical College, Weifang, China; ^20^ Department of Neurology, Shengli Oilfield Central Hospital, Dongying, China; ^21^ Department of Pediatrics, Affiliated Hospital of Jining Medical, Jining, China; ^22^ Department of Pediatrics, Tengzhou Central People’s Hospital, Zaozhuang, China; ^23^ Department of Neurology, Laizhou People’s Hospital, Qingdao, China; ^24^ Department of Pediatrics, Weifang Maternal and Child Health Care Hospital, Weifang, China; ^25^ Department of Neurology, Liaocheng People’s Hospital, Liaocheng, China; ^26^ Department of Pediatrics, Qilu Hospital, Cheeloo College of Medicine, Shandong University, Jinan, China; ^27^ Department of Neurology, Qilu Hospital of Shandong University (Qingdao), Qingdao, China; ^28^ Department of Pediatrics, Qilu Children’s Hospital of Shandong University, Jinan, China; ^29^ Department of Neurology, Affiliated Hospital of Binzhou Medical College, Yantai, China; ^30^ Department of Neurology, Dezhou People’s Hospital, Dezhou, China; ^31^ Department of Neurology, Linyi People’s Hospital, Linyi, China

**Keywords:** epilepsy, seizure, COVID-19, vaccine, safety

## Abstract

**Objectives:**

Several COVID-19 vaccines list “uncontrolled epilepsy” as a contraindication for vaccination. This consequently restricts vaccination against COVID-19 in patients with epilepsy (PWE). However, there is no strong evidence that COVID-19 vaccination can exacerbate conditions in PWE. This study aims to determine the impact of COVID-19 vaccination on PWE.

**Methods:**

PWE were prospectively recruited from 25 epilepsy centers. We recorded the seizure frequency at three time periods (one month before the first vaccination and one month after the first and second vaccinations). A generalized linear mixed-effects model (GLMM) was used for analysis, and the adjusted incidence rate ratio (AIRR) with 95% CI was presented and interpreted accordingly.

**Results:**

Overall, 859 PWE were included in the analysis. Thirty-one (3.6%) and 35 (4.1%) patients were found to have increased seizure frequency after the two doses, respectively. Age had an interaction with time. The seizure frequency in adults decreased by 81% after the first dose (AIRR=0.19, 95% CI:0.11–0.34) and 85% after the second dose (AIRR=0.16, 95% CI:0.08–0.30). In juveniles (<18), it was 25% (AIRR=0.75, 95% CI:0.42–1.34) and 51% (AIRR=0.49, 95% CI:0.25–0.95), respectively. Interval between the last seizure before vaccination and the first dose of vaccination (ILSFV) had a significant effect on seizure frequency after vaccination. Seizure frequency in PWE with hereditary epilepsy after vaccination was significantly higher than that in PWE with unknown etiology (AIRR=1.95, 95% CI: 1.17–3.24). Two hundred and seventeen (25.3%) patients experienced non-epileptic but not serious adverse reactions.

**Discussion:**

The inactivated COVID-19 vaccine does not significantly increase seizure frequency in PWE. The limitations of vaccination in PWE should focus on aspects other than control status. Juvenile PWE should be of greater concern after vaccination because they have lower safety. Finally, PWE should not reduce the dosage of anti-seizure medication during the peri-vaccination period.

## Introduction

COVID-19 has engulfed the world since its emergence in 2019 (1). According to data from Johns Hopkins University, 616 million people have been diagnosed with COVID-19, and 65 million people have died as of September 2022 (https://coronavirus.jhu.edu/map.html). In addition to being a public health crisis, COVID-19 has severely affected the global economy, agriculture, manufacturing, tourism, hospitality, and education to varying degrees (2). Today, COVID-19 continues to ravage the world, continually evolving into variants with increased infectivity (3). Universal vaccination remains the best way to fight COVID-19, and more than 9 billion vaccine doses have been administered worldwide, according to Johns Hopkins University (https://coronavirus.jhu.edu/map.html).

Patients with epilepsy (PWE) belong to a vaccine-restricted group. There are roughly 10 million PWE in China (4), and more than 70 million PWE worldwide (5). Restricted vaccination in PWE may lead to another COVID-19 outbreak due to these patients’ increased susceptibility. Moreover, “uncontrolled epilepsy” is listed as a contraindication for several approved COVID-19 vaccines in China. Although previous studies have shown that some vaccinations can induce febrile convulsions or seizures (6, 7), there is currently insufficient evidence that COVID-19 vaccination can induce seizures or exacerbate conditions in PWE. However, COVID-19 has the potential to induce seizures (8). Additionally, the pandemic’s effect on mental health and increased difficulty in seeking medical treatment will worsen the conditions of epilepsy patients (9, 10). Finally, some studies have demonstrated that PWE have a higher mortality rate compared to normal people (11–13); however, other studies have shown no significant difference (14, 15). These differing findings may be related to differences in PWE’s conditions and the anti-seizure medications (ASMs) used (some ASMs can interact with anti-COVID-19 drugs). But even in terms of patient protection, PWE are still greatly in need of the protection afforded by vaccination; after all, no studies have shown that epilepsy is a protective factor against COVID-19.

Our previous study showed that nearly 20% of PWE chose not to receive the COVID-19 vaccine due to concerns about possible adverse reactions (16). A similar study conducted in Lithuania reaffirms these findings (17). The safety of the COVID-19 vaccine continues to be questioned by PWE. Massoud et al. investigated adverse reactions in PWE following COVID-19 vaccination. They showed that most of the adverse reactions after vaccination were mild, with only a few PWE experiencing an exacerbation of seizures (18). Conversely, a previous retrospective study showed that approximately 16.8% of PWE experienced seizures after receiving the COVID-19 vaccine (19). Owing to the limitations of our previous study and the lack of high-quality studies that determine the impact of vaccination in PWE, we designed this prospective, self-controlled study with over 20 participating centers to discover the effects of COVID-19 vaccines and provide guidelines for COVID-19 vaccination in PWE.

## Methods

### Study design

From October 1, 2021, to January 31, 2022, patients diagnosed with epilepsy and scheduled to receive the COVID-19 vaccine were recruited from 25 epilepsy centers at 20 hospitals in Shandong Province, China. Informed consent was obtained from all participants or their guardians.

We did not oppose vaccination for all PWE except for those who had extremely frequent and unstable seizures and were of immunological origin. “Extremely frequent and unstable seizures” was defined as a history of seizure clusters (three or more seizures within 24h) or status epilepticus in one month before vaccination. As China limited the age of COVID-19 vaccination to over three years old, we only included PWE over three years old. And ultimately it was up to the vaccination agency whether PWE can administer the vaccine, as some PWE may have other contraindications. All PWE enrolled in the study were required to answer a two-part questionnaire. In addition to basic patient information, the first part included the etiology and duration of epilepsy, type of seizures (according to the criteria proposed by the International League Against Epilepsy in 2017). The first part was finished by PWE with the help of neurologists. The second part was completed by telephone or outpatient follow-up after vaccination and included current anti-seizure medications (ASM) and any adverse reactions, information about the vaccine, seizure frequency before and after vaccination, compliance with ASM during vaccination (all neurologists involved in the study were prohibited from prescribing lower ASM dose to their patients), and adverse reactions after vaccination and their severity. All PWE were required to keep diaries.

### Exclusion criteria

The exclusion criteria for the PWE were: 1) inability to complete follow-up visits or questionnaires; 2) concomitant with other conditions such as psychogenic non-epileptic seizures or unexplained syncope that affected the seizure judgment throughout the study period; 3) inability to provide accurate seizure history (including before and after vaccination); 4) failure to follow-up with patients or unwillingness of the patient to participate; 5) immunological causes of epilepsy.

### Vaccination

The primary vaccines in China are BBIBP-CorV (Beijing Institute of Biological products, Sinopharm, China) and CoronaVac (Sinovac Biotech Co Ltd products, Sinovac Biotech, China). Both the two vaccines require two doses. The interval between the two doses was generally 3-8 weeks. The enrolled PWE received the second dose at least 1 month after the first dose in case of statistical difficulties. All PWE enrolled received vaccine doses per the manufacturer’s regulations at designated medical facilities and adhered to post-vaccination precautions.

### Measurements

In this study, the outcome variable was seizure frequency. Baseline frequency was defined as the number of seizures within one month before the first vaccination. Seizure frequency after vaccination was defined as the number of seizures within one month after each vaccination. For an individual PWE, an increase, no difference, or decrease in seizure frequency was determined by directly comparing the numerical magnitude of frequency after vaccination to baseline frequency. If the seizure frequency of PWE after vaccination was higher/lower than the baseline frequency, an increase/decrease in seizure frequency was considered to have occurred after this dose.

The independent variables were age (adult vs. juvenile), sex, epileptic etiology (hereditary, structure, metabolism, infection, unknown), duration (years), number of ASM (zero, one, two, three or more), seizure type (focal, generalized, unknown), interval between the last seizure before vaccination and the first dose of vaccination (ILSFV) (less than 1 month, 1-2 months, 2-3 months, 3-6 months, 6 months to 1 year, 1-2 years, more than 2 years), medication status during vaccination (normal, reduction, withdrawal), and fever after vaccination (yes, no). This study did not include time-varying covariates.

### Statistical analysis

We determined the association between seizure frequency and explanatory variables using a generalized linear mixed-effects model (GLMM) with a Poisson error structure and log-link function and checked for over-dispersion in the data using R software version 4.0.3. First, we built a null model to analyze the cluster effects of “center” and “subject” on a random intercept. Second, we construct the random intercept model 1 with all potential fixed effects and selected the appropriate fixed effects by p-value (p<0.1) to construct a concise version (model 2). Third, we added a temporal random slope to model 2 (model 3) and select appropriate fixed effects by p-value (p<0.1) to further construct a complex model containing interaction terms (model 4-5). Akaike information criteria (AIC) and Bayesian information criteria (BIC) were applied for variable and model selection. Finally, we considered the GLMM with subject random intercept, time random slope, and time-age interaction as the better parsimonious model. The adjusted incidence rate ratio (AIRR) with 95% CI was presented and interpreted accordingly. Statistical significance was set at *P* < 0.05.

## Results

### Baseline characteristics

A total of 933 patients were recruited for the study. Of these, 66 were excluded from the analysis because they met the exclusion criteria. Eight patients did not wish to reveal their sex and were excluded because of missing values. Thus, a total of 859 patients were included in the analysis. Of these patients, 58.8% (n=505) were men, and 41.2% (n=354) were women. The median age of the participants was 20 (Q1:10, Q3:35), and the median duration of epilepsy was 4 years (Q1:2.5, Q3:9.5). Overall, 592 patients (68.9%) reported an unknown cause of epilepsy, while 267 (31.1%) reported a definite cause. Focal seizures were the most common seizure type (n=563, 65.5%) among PWE. The ILSFV in 376 patients (43.8%) was more than 2 years. Overall, 108 patients (12.6%) reported at least one seizure after vaccination. Forty-four (5.0%) and 39 (4.5%) patients reported seizures within 2 weeks after the first and second doses, respectively. Thirty-five (4.0%) and 39 (4.5%) patients developed seizures from three weeks to one month after the first and second vaccination doses, respectively. Thirty-one (3.6%) and 35 (4.1%) patients were found to have increased seizure frequency after the two doses, respectively. Most patients (n=516, 60.1%) were prescribed only one ASM, and 52 patients (6.1%) took three or more ASM. During vaccination, 27 (3.1%) patients had reduced ASM doses and 47 (5.5%) patients did not receive ASM. The specific baseline characteristics are shown in **Table 1**.

**Table 1:** Demographics.

Variables	N=859
Sex (n%)	
Male	505 (58.8)
Female	354 (41.2)
Age, year	
Median (Q1,Q3)	20.0 (10, 35)
Juvenile (<18) (n%)	408 (47.5)
Adult (≧18) (n%)	451 (52.5)
Median duration of epilepsy (Q1,Q3)	4.0 (2.5, 9.5)
Epileptic etiology (n%)	
Unknown	592 (68.9)
Heredity	89 (10.4)
Structure	147 (17.1)
Metabolism	2 (0.2)
Infection	29 (3.4)
Seizure type (n%)	
Focal	563 (65.5)
Generalized	191 (22.2)
Unkonwn	105 (12.2)
ILSFV (n%)	
Less than 1 month	94 (10.9)
1-2 months	42 (4.9)
2-3months	43 (5.0)
3-6 months	75 (8.7)
6-12 months	123 (14.3)
1-2 years	106 (12.3)
More than 2 yeas	376 (43.8)
Seizure after vaccination	
First dose (within 2 weeks) (n%)	44 (5.0)
Median of seizure frequency (Q1,Q3)	1(1, 2)
First dose (3weeks - 1 month) (n%)	35 (4.0)
Median of seizure frequency (Q1,Q3)	1(1, 1.5)
Second dose (withtin 2 weeks) (n%)	39 (4.5)
Median of seizure frequency (Q1,Q3)	1(1, 2)
Second dose (3weeks - 1 month) (n%)	35 (4.0)
Median of seizure frequency (Q1,Q3)	1(1, 2)
Total	108 (12.5)
PWE with increased seizure frequency (n%)	
First dose	31 (3.6)
Second dose	35 (4.1)
Total	61 (7.1)
Number of ASM (n%)	
0	42 (4.9)
1	516 (60.1)
2	249 (29.0)
More than 2	52 (6.1)
Medication status during vaccination (n%)	
Reduction	27 (3.1)
Withdrawl	47 (5.5)

ILSFV, interval between the last seizure prior vaccination and the first dose of vaccination; ASM, anti-seizure medications.

### Factors influencing seizure frequency

ILSFV was a strong predictor of the frequency of recent seizures. Compared to PWE who took regular doses of ASM, PWE who reduced the dose of ASM during the peri-vaccination period were found to have 98% more seizures with a statistically marginal difference (AIRR=1.98, 95% CI: 0.97–4.04, *P*=0.061). The frequency of PWE with hereditary etiology after vaccination was 1.95 times higher than that of PWE with unknown etiology (AIRR=1.95, 95% CI: 1.17–3.24, *P*=0.010). However, there was no interaction between etiology and time. Age was found to interact with time. In adults, seizure frequency decreased by 81% after the first dose (AIRR=0.19, 95% CI: 0.11–0.34, *P*<0.001) and 85% after the second dose (AIRR=0.15, 95% CI: 0.08–0.30, *P*<0.001) compared to baseline. After the first dose, the seizure frequency decreased level of adult is 3.69 times (AIRR=3.69, 95% CI: 1.83-7.45, *P*<0.001) as large as the seizure frequency decreased level of juveniles (<18). After the second dose, the seizure frequency decreased level of adult is 2.89 times (AIRR=2.89, 95% CI: 1.31-6.42, *P*<0.001) as large as the seizure frequency decreased level of juveniles. By constructing a model with juvenile as the age reference level, we found in juveniles, seizure frequency decreased by 25% (AIRR=0.75, 95% CI: 0.42–1.34, *P*=0.332) after the first dose and 51% (AIRR=0.49, 95% CI: 0.25–0.95, *P*=0.036) after the second dose compared to baseline. Detailed results are presented in **Table 2**. Differences in baseline characteristics between the two age groups are shown in **Table 3**.

**Table 2:** Risk factors of seizure frequency.

Predictors	Adjusted Incidence Rate Ratios (AIRR)	95% CI of AIRR	P
Time			
Baseline	Ref	–	–
First dose one month	0.19	0.11 – 0.34	<0.001
Second dose one month	0.15	0.08 – 0.30	<0.001
Age			
Adult	Ref	–	–
Juvenile	1.18	0.80 – 1.75	0.397
Number of ASM			
0	Ref	–	–
1	0.43	0.11 – 1.62	0.213
2	0.74	0.19 – 2.84	0.663
More than 2	0.56	0.14 – 2.28	0.415
Medication status during vaccinations			
Normal	Ref	–	–
Reduction	1.98	0.97 – 4.04	0.061
Withdrawal	0.66	0.17 – 2.59	0.556
ILSFV			
More than 2 yeas	Ref	–	–
Less than 1 month	424.83	173.48 – 1040.35	<0.001
1-2 months	14.67	5.04 – 42.65	<0.001
2-3months	10.08	3.31 – 30.64	<0.001
3-6 months	3.98	1.23 – 12.92	0.021
6-12 months	3.18	1.10 – 9.18	0.032
1-2 years	2.28	0.70 – 7.41	0.171
Etiology			
Unknown	Ref		
Heredity	1.95	1.17 – 3.24	0.010
Structure	1.28	0.82 – 2.02	0.280
Metabolism or Infection	1.14	0.53 – 2.47	0.738
Time*age			
First dose one month * juveniles	3.69	1.83 – 7.45	<0.001
Second dose one month * juveniles	2.89	1.31 – 6.42	0.009

ILSFV, interval between the last seizure prior vaccination and the first dose of vaccination; ASM, anti-seizure medications.

**Table 3:** Differences in baseline characteristics between adults and juveniles.

Variables	Adult (n=451)	Juvenile (n=408)	*P*
Sex (n%)			>0.999
Male	265 (58.8)	240 (58.8)	
Female	186 (41.2)	168 (41.2)	
Mean duration of epilepsy	10.79	3.92	<0.001
Epileptic etiology (n%)			<0.001
Unknown	291 (64.5)	301 (73.8)	
Heredity	14 (3.1)	75 (18.4)	
Structure	124 (27.5)	23 (5.6)	
Metabolism	2 (0.4)	0 (0)	
Infection	20 (4.4)	9 (2.2)	
Seizure type (n%)			0.004
Focal	312 (69.2)	251 (61.5)	
Generalized	80 (17.1)	111 (27.2)	
Unkonwn	59 (13.1)	46 (11.3)	
ILSFV (n%)			0.003
Less than 1 month	61 (13.5)	33 (8.1)	
1-2 months	25 (5.5)	17 (4.2)	
2-3months	26 (5.8)	17 (4.2)	
3-6 months	47 (10.4)	28 (6.9)	
6-12 months	62 (13.7)	61 (15.0)	
1-2 years	41 (9.1)	65 (15.9)	
More than 2 yeas	189 (41.9)	187 (45.8)	
Mean baseline seizure frequency	0.36	0.27	0.012
PWE with increased seizure frequency (n%)			N/A
First dose	8 (1.8)	23 (5.6)	
Second dose	14 (3.1)	21 (5.1)	
Total	21 (4.7)	40 (9.8)	
Number of ASM (n%)			<0.001
0	22 (4.9)	20 (4.9)	
1	236 (52.3)	280 (68.6)	
2	161 (35.7)	88 (21.6)	
More than 2	32 (7.1)	20 (4.9)	
Medication status during vaccination (n%)			0.786
Reduction	16 (3.5)	11 (2.7)	
Withdrawl	25 (5.5)	22 (5.4)	
Fever(n%)	3 (0.7)	9 (2.2)	0.079

ILSFV, interval between the last seizure prior vaccination and the first dose of vaccination; ASM, anti-seizure medications.

### Non-epileptic adverse reactions

**Figure 1:**
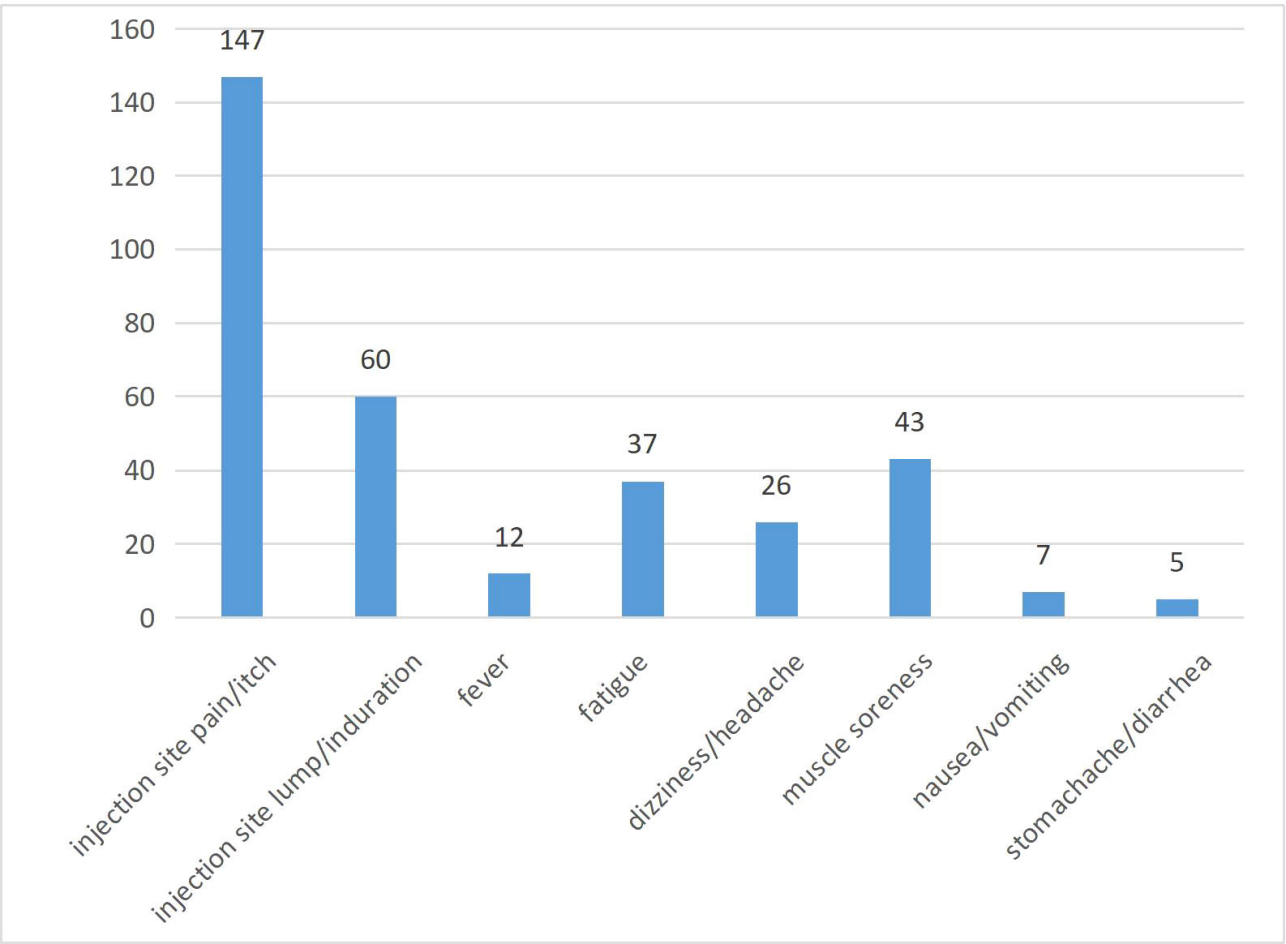
Non-epileptic adverse reactions.

Non-epileptic adverse reactions were recorded in all patients after the two vaccine doses were administered (**Figure 1**). Of 859 patients, 217 (25.3%) reported adverse reactions. Pain and itching at the site of vaccine administration were reported in 147 patients, resulting in two patients requiring medical attention. A lump and induration at the vaccination site occurred in 60 patients, resulting in one patient requiring medical attention. Nine patients reported low fever (37.1-38.0°C), and three reported moderate fever (38.1-39.0°C). Thirty-seven patients experienced varying degrees of fatigue. Twenty-six patients reported varying degrees of dizziness and headache, one of whom required medical attention. Muscle soreness occurred in 43 patients, with one patient requiring medical attention. An additional 11 patients developed gastrointestinal symptoms, including nausea, vomiting, stomachache, and diarrhea. None of the 859 patients developed serious non-epileptic adverse events such as Guillain-Barre syndrome or anaphylactic shock.

## Discussion

In this prospective, multicenter, self-controlled trial, seizure frequency tended to decrease after vaccination in both adults and juveniles, although the magnitude of the decrease varied. As a continuation of a retrospective study, this study provides further evidence that inactivated COVID-19 vaccination does not affect non-immune epilepsy, and epilepsy should not be a contraindication for vaccination.

Similar to our previous study, 108 PWE (12.6%) experienced seizures after either the first or second vaccine dose in the present study (19). In these patients, epilepsy control conditions before vaccination were not identical, and several patients reported frequent seizures within one month before vaccination. Therefore, the occurrence of seizures is not an accurate indicator for this population, and the seizure frequency in PWE before and after vaccination was used. Overall, 61 patients (7.1%) were found to have increased seizure frequency after at least one of the two doses, significantly lower than the rate of seizures after vaccination. Further analysis showed varying degrees of reduction in seizure frequency after each of the two doses compared to the baseline. Previous studies have shown that seizures within 14 days after other types of vaccination may be vaccine-associated (6, 7, 20). Consequently, our observation period was extended to 30 days; however, it did not show a significant increase in seizure frequency compared with the baseline frequency and instead showed a significant decrease. This significant reduction may have resulted from the normal physiological phenomenon rather than vaccination, as neurologists may have adjusted ASM regimen when PWE were enrolled in the study, especially in those with frequent seizures. In addition, the baseline frequency was probably recorded as the only seizure in a given seizure cycle when seizures stabilized after proper treatment. Therefore, this study’s “time” effect can be explained as an effect of the medical intervention and disease characteristics during the particular period. This suggests that COVID-19 vaccination does not worsen the condition of PWE under a rational medication regimen. Notably, the decline in frequency was more pronounced in adults than in juveniles. This may be due to a difference in baseline frequency. Adult PWE have a higher baseline frequency, which allows for a more notable decline in seizure frequency after possible drug adjustment.

In conclusion, the most important implication of this study is that inactivated COVID-19 vaccination did not increase seizure frequency in the vast majority of PWE. Currently, “uncontrolled epilepsy” is a contraindication for many vaccines; however, vaccine manufacturers do not have clearly defined parameters for “uncontrolled epilepsy.” Expert guidelines in China recommend that PWE should not consider vaccination until they have been seizure-free for more than 6 months. However, our previous studies have demonstrated that a seizure-free period of over 3 months would not significantly decrease the risks associated with vaccination (19). This study further expands the indications for vaccination in PWE by demonstrating that control status should not be a limitation for vaccination. Another important finding was that juvenile PWE were less safe after vaccination than adult PWE; after the first dose, the seizure frequency did not differ significantly from baseline. Our results are in agreement with the studies by Lu et al. and Özdemir et al., which found a low rate (< 10%) of seizure exacerbation in PWE after vaccination (21, 22). Similar results were reported in a meta-analysis done by Zheng et al. (23) Our finding that set our study apart from a few other studies was that the seizure frequency of PWE in different age decreased to different degrees after vaccination. However, even if vaccination was safe for juvenile PWE, the vulnerable group should be observed rigorously after vaccination, especially after the first dose. Finally, adult PWE should be encouraged to get vaccinated against COVID-19.

As a neurotropic virus, mediated by neuropilin-1 (NRP-1) or angiotensin-converting enzyme 2 (ACE2) (24, 25), SARS-COV-2 can invade the central nervous system (CNS). SARS-COV-2 may severely hamper cerebral homeostasis upon entry into the CNS by destroying the blood-brain barrier (BBB) and glial limiting factor, activating the toll-like receptor (TLR), and ultimately leading to neuronal death (26). Several studies have reported that activation of TLR may promote epilepsy (27, 28), while others have reported that immune cell and serum protein infiltration due to BBB destruction also exacerbates epilepsy (28, 29). Moreover, SARS-COV-2 entry into the CNS could hamper the respiratory regulation center in the brain stem (30), which would significantly increase the risk of sudden epileptic death (31), and potentially contribute to increased mortality in SARS-COV-2 infected PWE. Aladdin et al. and Šín et al. have also reported two cases of status epilepticus due to the COVID-19 vaccine (32, 33); however, one patient was vaccinated with an mRNA vaccine and one with an adenovirus vector vaccine. In addition to preventing infection, the COVID-19 vaccine can also reduce the illness of those infected with SARS-COV-2 (34, 35), which is particularly important for neurologists, as those with severe infection are more likely to develop neurological symptoms than those with non-severe infection (36). In addition, SARS-COV-2 can enter the CNS directly through the BBB (26). Some studies have found that the BBB of PWE has different degrees of destruction (37), which may lead to PWE experiencing more CNS attacks after infection with SARS-COV-2. This may then aggravate epilepsy or cause other neurological diseases, worsening the condition of PWE.

The PWE recruited in the present study reported a lower rate of non-epileptic adverse reactions. Of the 859 patients, 217 (25.3%) had various non-epileptic adverse events, and none had severe non-epileptic adverse events. Currently, WHO-approved COVID-19 vaccines include RNA, adenovirus vector, and inactivated vaccines. Mass vaccination in China primarily uses inactivated vaccines produced by Sinopsin and Sinovac; therefore, most PWE enrolled in this study received inactivated vaccines. When given inactivated vaccines, the rate of non-epileptic adverse reactions was significantly lower than that of the normal population after adenovirus vector and mRNA vaccination (38, 39). A meta-analysis by Cheng et al. showed that all three vaccines provided effective protection against SARS-COV-2 infection; however, the efficacy of inactivated vaccines was the lowest, whereas their safety was the highest among the three vaccines (40). A study by Xia et al. reported adverse reactions in 29% of the Chinese population, which received inactivated COVID-19, and an absence of any severe adverse reactions (41). Therefore, since a majority of our cohort received inactivated vaccines, a reduced rate of adverse reactions was attributable to the safety of these vaccines. In addition to the safety of inactivated vaccines, side effects associated with COVID-19 vaccination in PWE may be mistaken for the adverse reactions caused by ASM, resulting in a lower rate of non-epileptic adverse reactions following vaccination.

Although existing studies have shown that inactivated vaccines are safer, mRNA vaccines have been shown to have higher immune efficacy. Since PWE may be more susceptible to mortality following infection with COVID-19 (8, 11–13), the type of vaccine to be administered should be determined by assessing the local epidemic situation. In the case of elevated local infection risk, the mRNA vaccine should be administered, whereas the inactivated vaccine should be administered when the local risk is lower.

Although the results showed that COVID-19 vaccination did not lead to a significant increase in seizure frequency, several patients, who had been seizure-free for over two years and were not using ASM, reported seizures during the vaccination follow-up. As with new-onset status epilepticus caused by COVID-19 vaccination, this may be incidental. Lamberink reported that an abnormal electroencephalogram (EEG) was a risk factor for seizure recurrence in PWE after drug withdrawal (42). Consequently, we recommend that these PWE undergo an EEG; PWE with an abnormal EEG should re-consume ASM as per the previous regimen around the vaccination period (one month after each dose) to prevent a recurrence.

Additionally, we found three notable factors that did not interact with time. First, the seizure frequency in patients with hereditary epilepsy after vaccination was higher than that in PWE with unknown etiology, which may be related to the fact that PWE with hereditary etiology were mainly juveniles (84.3%), while only a small proportion of adult PWE (15.7%) had hereditary etiology. As mentioned above, the decrease in seizure frequency after vaccination was less pronounced in juveniles than in adults. Second, ILSFV was also found to be a predictor of seizure frequency after vaccination. Although PWE with short ILSFV also experienced a decrease in seizure frequency after vaccination, this decrease may be insignificant in the context of its own higher seizure frequency. Therefore, in consideration of the condition, PWE with short ILSFV (especially shorter than three months) still need to be paid attention to during the peri-vaccination period. For example, if fever occurs after vaccination, PWE should be treated in a timely manner. In addition, PWE should increase the frequency of visits to the neurology department after vaccination. Finally, neurologists may consider appropriately increasing the dosage of ASM during the peri-vaccination period, which can not only prevent vaccine-induced seizures but also help the PWE’s existing condition. Third, given that the factor of ASM reduction during vaccination was at a statistical critical point (AIRR=1.98, 95% CI: 0.97–4.04), even if epilepsy is well controlled, we do not recommend reducing the dosage of ASM before or after vaccination.

This study had certain limitations: the majority of PWE received inactivated vaccines, thus limiting significant references for other COVID-19 vaccines; the study did not include PWE with immune etiology since it would not be possible to determine the impact of COVID-19 vaccination; the highest seizure frequency in this study was 24/month, so the results may not apply to PWE with frequent seizures; and information about seizures relied on the reports of PWE, which may affect the accuracy of the data due to subjective errors.

## Conclusion

In summary, this study demonstrated that inactivated COVID-19 vaccination does not significantly increase seizure frequency in patients with non-immune epilepsy, and the local infection risk should be considered when using inactivated COVID-19 vaccines in PWE.

## Data availability statement

The original contributions presented in the study are included in the article/supplementary material, further inquiries can be directed to the corresponding author/s.

## Ethics statement

The studies involving human participants were reviewed and approved by Medical Ethics Committee of Qilu Hospital of Shandong University. Written informed consent to participate in this study was provided by the participants’ legal guardian/next of kin. Written informed consent was obtained from the individual(s) for the publication of any potentially identifiable images or data included in this article.

## Author contributions

XueL and XF had the idea for the study. XueL, XF, and TH were responsible for study design. XF, TY, JH, YSo, CL, AM, YLi, QK, LT, WC, WXS, CF, YSu, JC, WYS, YY, YG, JG, NL, QL, ZL, SL, WLa, XinL, SW, YC, BL, LL, XiaL, YLiu, YZ, ZG, LQ, QF were responsible for data collection. XueL, XF, and TH were responsible for coordinating and supervising the work of each center. XueL, XF, and SH were responsible for data analysis, data interpretation, writing, and reviewing of the manuscript. All authors agree to be accountable for the content of the work. All authors contributed to the article and approved the submitted version.
