# Supplementary material for: Effect of inactivated COVID-19 vaccines on seizure frequency in patients with epilepsy: A multicenter, prospective study
Source: Front Immunol. 2022 Dec 7;13:984789. doi: 10.3389/fimmu.2022.984789 (PMC9769399; doi:10.3389/fimmu.2022.984789)
Supplement: Supplementary file 1 [file DataSheet_1.pdf]

Table S1 Selection of Random Effects and Fixed Effects to be Included in the GLMM

Model	Random Effects and Fixed Effects	AIC	BIC	Deviance
	~1+(1|subject)*	2006.9	2018.6	2002.9
1	~ time+age+sex+ASM+duration+seizuretype+MedicationStatus+ILSFV+fever+etiology+(1|subject)	1568.1	1708.6	1520.1
2	~ time+age+ASM+MedicationStatus+ILSFV+etiology +(1|subject)	1562.1	1673.3	1524.1
3	~ time+age+ASM +MedicationStatus+ILSFV+etiology+(time|subject)	1443.1	1583.6	1395.1
4	~ time+age+ASM +MedicationStatus+ILSFV+etiology+time:age+(time|subject)	1431.1	1583.3	1379.1
5	~ time+age+ASM+MedicationStatus+ILSFV+etiology +time:etiology +(time|subject)	1451.7	1627.3	1391.7

* In the null model step, the Intraclass-Correlation Coefficient (ICC) of "subject" is 0.76, however, the "center" variable did not show any cluster effect. We only keep "(1|subject)" as random intercept.

Table S2 Seizure frequency in three time periods of PWE with different etiologies

Variables	*Mean Baseline frequency*	*Mean Frequency after first dose*	*Mean Frequency after second dose*
Etiology (SD)			
Unknown	0.26 (1.12)	0.14 (0.68)	0.15 (0.78)
Heredity	0.57 (2.82)	0.24 (0.68)	0.20 (0.55)
Structure	0.37 (1.64)	0.12 (0.54)	0.10 (0.48)
Metabolism	0 (0)	0 (0)	0 (0)
Infection	0.48 (1.41)	0.1 (0.31)	0.1 (0.31)
